# Supplementary material for: Predicting host taxonomic information from viral genomes: A comparison of feature representations
Source: PLoS Comput Biol. 2020 May 26;16(5):e1007894. doi: 10.1371/journal.pcbi.1007894 (PMC7307784; doi:10.1371/journal.pcbi.1007894)
Supplement: Attachment — Submitted filename: Young-etal-response-reviewers.pdf [file pcbi.1007894.s007.pdf]

Submissions
PLoS Computational Biology

Dear Drs Pitzer and Langille,

Thank you for the opportunity to resubmit our manuscript ‘Predicting host taxonomic information from viral genomes: a comparison of feature representations‘. In our response to the reviewers below we describe the changes to our text and provide specific responses to the three reviewers. The main changes are we have re-written our text to make the focus of the research clearer, added discussion on our choice of machine learning approach and its rationale, and added more biological interpretation where appropriate. In response to reviewer 2 we have added some additional analysis: (i) we have carried out further work to address the issue of using the ICTV taxonomic tree to select groups of phylogenetic related viruses for our holdout groups. Specifically, we have added a filtering step using average nucleotide identity ANI to remove any viruses related to the holdout group from the training data. The results of this have replaced our original holdout results. And, (ii) to check if our features contained complementary signals we tested combining features from the different representation use a kernel combination method. As a result of the referees’ input, we believe our manuscript is much improved and we thank the reviewers for their helpful comments.

We’d like to emphasise that our intention was to investigate the potential of a wide range of features for use in ML approaches to VH prediction. Rather than aiming to produce a tool optimised to a specific problem, our aim was to compare a large number of different feature sets, and compare their predictive power over a large number of datasets. We have successfully shown that there is merit in using these features in prediction. Extracting these features from the viral genomes is not difficult, with many bioinformatics tools available (and the code used by us is provided on GitHub). The difficulty in developing a useful predictor is optimising all the options to the specific task in hand. This would need to be done on task by task basis with experts in the particular domains involved and so beyond the scope of this work.

We hope you now find our manuscript acceptable for publication in your journal. Please don’t hesitate to contact us if you require any further information. We look forward to hearing from you.

Yours sincerely,

David L Robertson, PhD
MRC-University of Glasgow Centre for Virus Research (CVR)

Reviewer's Responses to Questions

Reviewer #1:

Thank you for the many constructive suggestions for improving our manuscript. In particular we appreciate the time and detail you have put in to identifying our mistakes. Below we have addressed all your points as best as possible.All our responses are in blue with text from the manuscript in quotes and italics, and with new or altered text in blue.

This study compares a range of sequence-based signals for virus-host prediction. While the analysis potentially has merit, I have several comments.

Some parts of the text are unclear and should be rewritten/explained better:

> Thank you for suggestions for improving the text. We have re-written or added to the text to improve clarity:

- line 67-69

> We have added:
>
> "*Di-nucleotide features , in particular, have been included in a wide range of virus host prediction tasks, from training on a single virus species or genera with multiple hosts such as rabies virus, coronavirus, and influenza A virus, [14–16] to training on host taxa with multiple viruses [17–19].*"

- line 134

> We have added:
>
> *"By setting a low threshold of 28 as the minimum class size, giving a total dataset size of 56, we were able to include more datasets at species and genus level"*

- It is unclear what the 120 datasets are (line 143). It might be good to present a Supplementary Table listing all these datasets with the number of viruses.

> Yes we agree, please see Supplementary Tables 1 and 2

- line 147: "extracting the 20 different feature set matrices" - which 20? That is unclear at this point and might be presented in a Table.

> We have moved Table 1 from Methods to Results to increase clarity.

- In the family holdout analysis it is unclear which families are held out.

> This is now included, see Supplementary Table 3

- line 267-268

> We have re-written this sentence:

“In a minority of cases there was a complete loss in predictive power implying the classifier trained on all the viruses must be using signal specific to the holdout group which was will not be present in the training set of the holdout classifiers.”

-"suggesting a host-specific signal" (line 270-271): explain better, please don't cut corners

We have added an explanation of host-specific signal:

"a host specific signal, that is a common signal that is specific to viruses that infect the labelled host."

- line 282-284: unclear why would this decrease the ratio?

The ratio will decrease because common domains are more likely to co-occur in phylogenetically related viruses than be due to convergence as it is rare, but not unknown, for domains to form through convergent evolution. Therefore by removing the phylogenetic signal from the holdout datasets we should expect a large drop in prediction as this is the signal we aimed to remove.

We have re-written the text to improve clarity:

“Convergence of domains is rare but they remain evident in diverging genomes and therefore will be more likely to contribute to the phylogenetic element of the signal which has been removed. Cases where the domain signal is not lost maybe indicate distant phylogenetic relationship or be due to common domains arriving via HGT.

- I don't follow the argument in line 317-319. Where did you look at synonymous mutations?

Apologies for any confusion, we have re-written this text:

“Interestingly, even though using k-mers will lose information due to single mutation in sequences, prediction increases for longer k-mers of the nucleotide sequences meaning it does not appear to be affected by the high mutation rate that occur within viruses. This is in accordance with HostPhinder[37] successfully used co-occurring k-mers of length 8 to predict host and the finding that even after controlling for HGT, much long nucleotide sequences co-occur in viruses and their host across all classes of viruses and host Goz [38]."

- It is unclear what they mean by "precise local virus-host molecular interactions" (e.g. line 321)

We have re-written to add more explanation to make this clearer:

"Longer k-mer features and domains - which only occur once or a few times in a genome - have the capacity to encode information about local virus-host molecular interactions, such as motif-domain or domain-domain interfaces. This is opposed to the shorter oligio-nucleotides which occur multiple times in a genome and therefore gives a global measure of biases over the whole genome.”

- what does "This" refer to in line 331?

We have added ‘data’, for clarity." *This data is biased”*

- unclear why "these false negatives will occur more frequently at higher taxonomic ranks" (line 337)

We have re-written this to include a clearer explanation and corrected higher to lower taxonomic ranks:

"Secondly, the negative data are viruses that are not known to interact with the host and may include viruses for which interactions that have not yet been unobserved. Because viruses tend to infect closely related hosts these false negatives will occur more frequently at lower taxonomic ranks."

- line 411-412

We have re-written this sentence to improve clarity:

"For each genome in a dataset the domain composition vector was made by counting the frequency of the unique domains in each virus across the set of all unique domains found within that dataset. These vectors were then normalised to sum to 1."

I think the authors may be new to the field as there are several inaccuracies throughout the text:

- line 51 & 361 "orphan genes" are viruses that are not associated with a disease but may possess pathogenicity (wikipedia)

We have removed 'orphan' from both lines.

- ref 4 is not the ref for IMG/VR, which should be Paez-Espino et al. 2016 NAR 45:D457 or Paez-Espino et al. 2018 NAR 47:D678.

Apologies we now reference Roux (2019) for the 5% figure and Paez-Espino et al. 2018 NAR 47:D678 for IMG/VR.

- viral tagging (ref 5) is not a "low throughput experimental method". It is also unclear how "reliable" it is (line 53).

We have removed this reference.

- The study may benefit from collaboration with a taxonomist, as some terms are confused. For example, the authors consistently mix up high and low taxonomic ranks, and "taxon group" is unclear in line 381 (this should probably be" parent"?).

We have removed all of the inconsistencies with reference to taxonomic rank throughout the text.

line 381 has been changed to: *"that infect the rest of the hosts in its parent node"*

- They repeatedly set up a straw man that only short nucleotide k-mers have been used until now, e.g. lines 64, 297, 313. This is annoying and does not do justice to a lot of literature, for example see refs 29, 30 (and others) and Edwards et al. 2016 FEMS Microbiol Rev 40:258.

We have added emphasis throughout the manuscript that the focus of this study was to make a comparison of the predictive power of features specifically for machine learning. To place this study in context to other computational approaches to virus host prediction, we have added an overview of current non-machine learning

methods including k-mer composition similarity comparison and have included these 3 references to the introduction:

"Computational approaches to virus host prediction fall into four broad strategies: searching for homologous sub-sequences in the hosts, such as prophage [5] or CRISPR-Cas spacers [7]; looking for co-abundance between virus and host [8]; comparison of oligonucleotide or k-mer composition either with potential host genomes [7,9,10] or with a reference virus genomes [11]; or machine learning approaches as described below. Although the first strategy can give high confidence predictions they are constrained the limits of alignment approaches at low sequence similarity. K-mer profile comparison provide alignment free methods but distance metrics lack of contrast when measuring proximity in high dimensional space and hence lose discriminative power. Additionally all reference based methods are constrained by the genomes available in the databases."

We think we are justified in stating that the majority of machine learning methods have used short nucleotide k-mers. In the current literature on machine learning approaches all but 2 of the methods use nucleotide features, and over half use di-nucleotides. We have discussed these two methods the alternative features used by Raj and Leite in the introduction (line 89-92), and in the discussion (lines 303-304), and the use of longer kmers used by Zhang et al. (2017). On this we have added the following to the text:

64 -"*To date, most machine learning approaches to virus host prediction have used features derived from oligeo-nucleotide …"*
In this paragraph we are talking about machine learning approaches, all but 2 of the methods use nucleotide features.

297 -"*The majority of previous approaches to virus-host prediction have focused on information from the nucleotide sequences…"*
Again in this paragraph we are talking about genome representation, all but 2 of the methods use nucleotide features.

313.- In this paragraph in the Discussion we are talking about the impact of k-mer length on prediction, over half the machine learning approaches use di-nucleotides.

We have substantially changed this paragraph and have included references to other computational approaches.
"*Although many machine learning approaches have used di-nucleotide features[18–22], other computational approaches have shown that using longer k-mers (length 6 and 8) is beneficial to prediction [9–11]. Zhang et al, (2017) found that with random forest classification increasing nucleotide k-mers up to a length of 8 improved prediction."*

The overlap between training and testing data is not sufficiently discussed. This also affects many of the previous assessments. While the family holdout analysis addresses this to some

extent, it is not discussed anywhere how this affects host predictions for new viruses from metagenomes that have not been seen before.

There is no overlap between training and test data. We have added this explanation to the results so this is clearer:

"To ameliorate the problems caused by overlapping or redundant data we aimed to keep a minimum distance between sequences by only including one genome sequence per viral species, the reference sequence."

Although this is not an ideal solution as the distances between species across different viral taxonomic groups is highly inconsistent we are not trying to claim a definitive accuracy across all our datasets but rather demonstrate that our features are predictive.

We have also added the use of average nucleotide identity ANI, as suggested by Reviewer 2, to filter closely related viruses from the training data in our 'holdout' datasets. This addresses the issue about the potential for prediction for new viruses. While there are no guarantees that classifiers will successfully predict the host of newly discovered families of viruses, the holdout results show we can predict for phylogenetically related groups of viruses not included in the training dataset.

Related to the point above, it is worrying that longer k-mers perform better and unsatisfying that they stopped at k=9. This points to overlap between training and testing data (redundant viruses).

We addressed this interesting point in the discussion:

"Interestingly, even though we might expect longer k-mers to perform badly due to the presence of mismatches, prediction increases for longer k-mers and therefore does not appear to be affected by the high mutation rate that occur within viruses. This is in accordance with HostPhinder[11] that successfully used co-occurring k-mers of length 16 to predict hosts and the finding that, even after controlling for HGT, much longer nucleotide sequences co-occur in viruses and their host across all classes of viruses and host [46]."

We have also addressed the reason we stopped at k=9 in the methods section (line 419):

"The maximum length of k-mers at each genome representation level was chosen to restrict the feature set size and keep the workflow computationally reasonable."

There are over 4 million possible DNA kmers k=10 kmers and 3 million AA kmers of k=6. This leads to vastly increased compute time and memory usage so as to become untenable for this number of datasets. These large feature matrices becomes very sparse and a more suitable algorithm should be used.
You can also see that the incremental improvement with increasing k is decreasing which would mean that the payoff for improved prediction against the increased compute time is reducing. At the point of building a predictor for a specific task it may be worth testing the limits of this improvement.

While the family holdout isolates the" host specific signal" to some extent, they did not isolate the" phylogenetic signal" and can thus not conclusively say that the difference in prediction performance fully represents this signal. This should at least be discussed.

We agree that using the viral taxonomy as a substitute for phylogeny is incorrect but due to the lack of a phylogenetic tree for all phage we hoped this would be a reasonable proxy given that in general phage genera appear to represent distinct clades of viruses. Following a suggestion from reviewer 2 we have now addressed this in the holdout method. By using average nucleotide identity,ANI, as a measure of genetic relatedness to remove viruses with >75% ANI to the holdout group from the training set. A description and discussion of this is included in the methods and the results.

The analysis uses only complete viral genome sequences, but metagenomics often yields incomplete genomes. It should be discussed what is the expected effect of this.

This study is focused on comparing the potential of features for use in a wide range of virus host prediction tasks not to develop a predictor for a specific tasks. As with all machine learning classifiers, they need to be developed and optimised for a particular task. For contigs of reasonable length we would expect the predictions to be reliable.

We have added the following text to the discussion section:

“It will be important to test the effects of partial or incomplete genomes on the performance of the classifiers to ascertain the usefulness of these features for use in metagenomics.“

They mention that" One reason for this drop in predictive power and increased variance is the decrease in size of the datasets" (line 213, 335). This can be easily tested by selecting the same amount of training data at all levels.

Whether or not increasing the number of training examples will improve performance depends on the particular prediction task that is being carried out. In particular, how separable the two classes are and the diversity of the training examples. We have added an illustration of this to the discussion:

“The lack of correlation between the size of the dataset and score is very apparent at geneus and species level, (Fig 2), with the classifiers for the datasets S.enterica (192 viruses), E.coli (465 viruses) and the genus mycobacterium (262 viruses) performing particularly badly across all feature sets.“

While they controlled for" a minimum number of virus species" (line 376) the diversity of these viruses is not taken into account

It would be very interesting to study the effect of virus diversity on the accuracy and of the generalisability of predictions, but due to the difficulty in comparing virus diversity at deeper levels of evolution than species this would be a considerable amount of work and beyond the scope of this paper.

I can't find anywhere which" multiple" families were held out in the family holdout analysis (line 246).

This is now included as supplementary Table 3

Could the" genetic signature that are [IS] shared across all virus families including the holdout 'family'" (line 272) be due to HGT?

This is a very good question. HGT is definitely a potential explanation, but we think it is less likely that the signal would remain in the sequence data more strongly than in the domains across whole phyla of bacteria. However, we see a larger signal loss in the domain datasets. Due to the rapid rate at which HGT units amerilorate to their host's genomes we feel that the nucleotide or amino-acid k-mer composition will be more affected in more distant transfer events as compared to the domains which are conserved for much longer. Another source for the signal may come from the co-occurring sequences (median length 40bp) in the virus and hosts genomes even after controlling for HGT which could also be the source of the signal as found by Goz et al 2018.

We are very interested in understanding the origin of the different aspects of these signals and are carrying out in depth investigations to investigate the role of HGT. However, due to the extensive nature of the datasets used this is beyond the scope of this paper.

We have added this possibility to the discussion that this may be due HGT:
Cases where the domain signal is not lost maybe indicate distant phylogenetic relationship or be due to common domains arriving via HGT.

You did not" limited this study to using kmer composition" (line 343) as you also looked at domains.

This is correct and made it clearer domains were also looked at. We have changed line 343 to make this point more explicitly about the sequence representations we used.
"*We have limited this study to using kmer composition of the sequences.*"

It is unclear why 28 was the minimum size of the positive set. It is also unclear where the 56 comes from (line 377), but I am assuming this is 28 positives + 28 negatives. It is also unclear how the total number of viruses in the dataset could be" less than 50" (line 397) of the minimum number is 56.

28 was chosen as it represented a trade-off between the number of viruses in a data set and the total number data sets that could be included. At line 377-379 we have added a clearer explanation of our choice of 28 as our minimum class size:
"setting this arbitrary threshold low enabled us to include more examples of genus and species level datasets."

You are correct the minimum dataset size of 56 was based on the positive and negative class size of 28, we have now stated this at the end of the paragraph:
"This resulted in binary datasets of equal numbers of positive and negative viruses with a minimum dataset size of 56."

Line 397- This was an error where we are talking about increasing the ratio of test to training data for the smaller datasets, we have now altered this:
"dataset less than 100 ."

If the above is correct, this means that the P:N ratio was 1:1 which is not realistic. In nature we expect a huge class imbalance. This should be addressed and/or discussed.

Our goal in this study was to assess the predictive power of different feature sets. As such, we made the decision to balance all datasets to remove the influence in performance that imbalance would have. Of course, such imbalance would need to be taken into consideration when training (and defining operating thresholds) in a deployed system.
We have added the following text to the discussion.
"This optimisation could include some measure of the prevalence of the data to take account of class imbalances."

It is unclear how the k-mers were extracted. The method should screen both the leading and lagging nucleotide strands and/or reverse complement k-mers should be accounted for.

We used our own code to extract the k-mers from the different sequences.To improve the clarity of our Methods we have:
i) added an extra sub-heading heading in the methods:" *K-mer extraction*"
ii) improved the description of how we generate a k-mer composition matrix in the Methods section, sub-section 'K-mer extraction':"*All sequence data was represented as a vector of k-mer composition. These were generated by counting the number of times each possible k-mer occurs within the sequence (and the reverse complement of the nucleotide sequences), and then normalising the resulting vector to sum to 1 to account for varying genome lengths.*"

Concatenating segments leads to false k-mers overlapping the concatenation site (line 400). This should be avoided.

We agree that concatenation leads to false k-mers but the number of erroneous k-mers ((number of segments -1) *(k-1)) will be very small as compared to the total number of k-mers (length of genome-k+1). We believe that although there is potential for a small improvement in prediction, SVM is able to cope with this small amount of additional noise. This study was about exploring and comparing the predictive potential of different features and at no point were we trying to optimise prediction.

Settings ad cutoffs of hmmscan are not mentioned. It is unclear how many domains were found.

We have added the cutoff settings to the methods:
"*The Hmmscan setting: --cut_tc option was used,"*
Number of domains: The number of unique domains found in all the viral genomes (2200) is recorded in Table 1.

Figures:

Thankyou for your suggestions for improving the interpretability of the figures, we hope we have addressed all your points.

- In the heatmaps it might be insightful to indicate next to the rows how large the datasets were.

We have included the dataset sizes in the axis labels of the heatmap figures in supplementary

- In Figure 3 what are the rows?

Due to the number of rows in this heatmap and the restriction in the size of the figures we are unable to include row labels. We have included a fully annotated heatmap, including dataset sizes, in the supplementary data.

- It might be good to provide a Supplementary Table with the actual numbers and refer to it in the fig legend

Please see Supplementary tables S1 and S2

- Figure 6 is confusing: there are red, green and orange boxes, the word" holdout" is mentioned twice, and there seems to be overlap between training and testing. There is no scale for the purple shading.

We have redawn the figure and added numbering to help clarify the approach being used. This figure is a schematic representation of how we constructed our holdout datasets and as such the virus host interaction matrix was meant to be representative only. It is demonstrating how the holdout group were removed before making the training set, therefore there is no overlap in the two datasets.

- Figure 7A is turned 90 degrees relative to the others. It is difficult to make sense of the order of rows (columns).

We have rotated the figure to match the other heatmaps and added the dataset label holdout and dataset size to the x-axis labels.

- What is the arrow in Figure 8?

This has been removed.

The manuscript is very sloppily written and the authors might consider professional editing. Just some of the many examples:

We apologise for the errors in the text and thank you for your time in pointing them out,. Below we have indicated how we have addressed each point:

-"keep up with the rapid pace with viral discovery" (line 54)

We replaced" with" with" *of"*

- words like" literally" and" actually" do not belong in scientific text

We removed:" literally"

We replaced" actually" with" *in effect"*

- repeated words (line 170)

We removed duplicate.

- mistakes with interpunction (line 186)

Corrected

- They say an AUC of 0.92" compares with" an AUC of 0.64 (line 211-213) but this is a huge difference. It is unclear to me where those numbers came from as no figure is referenced.

We have *r*e-structured wording to improve clarity:

"For the bacteria datasets at phylum level all the feature sets (with the exception of DNA k=1), Figure 4.a.i, are highly predictive with an average AUC of 0.86 and standard deviation of 0.07. Whereas the species level classifiers have an average AUC of 0.67 and standard deviation of 0.15, Figure 4.a.vi"

- Present/past tense mixup (line 249)

We changed, choose to *chose*.

- Inconsistencies (Fig. vs Figure, kmer vs k-mer)

We replaced all Fig with *figure*.

We replaced all kmer with *k-mer*.

- long and convoluted sentences (line 276)

We re-structured the sentence to improve clarity:

It is difficult to identify a consistent pattern as to which feature sets have the biggest signal loss, although protein domains (Domains_1), and physio-chemical property derived features (PC_5, PC_6), have more datasets where the holdout classifiers have a big signal loss (Figure 7b). While, for the majority of datasets these features remain predictive, roughly a quarter of the datasets have a large drop in AUC, with ratios of less than 0.75. As a comparison, none of the DNA_6, DNA_9 and AA_3 datasets had a ratio of less than 0.75.

- sentence structure (line 329)

We added-"*training examples, hence we are"*

- Supplementary X (line 406)

Removed - redundant information.

- Domains -The (line 407)

We have re-written this -"*Domain content for each genome was identified"*

- They cite the user manual of HMMER.

We have replaced the incorrect citation, Eddy SR. Accelerated Profile HMM Searches. PLOS Comput Biol. 2011;7: e1002195. doi:10.1371/journal.pcbi.1002195

Reviewer #2:

Thank you for your many constructive comments. We have incorporated your suggestions into our manuscript including testing for feature redundancy and using ANI to filter phylogenetically related viruses from the training data in the holdout datasets. All our responses are in blue with text from the manuscript in quotes and italics, and with new or altered text in blue.

In this manuscript, the authors aim to study the" host" information embedded into viral genomes. To that end, the authors explore and describe the predictive power of different genome features across viral taxonomy, using AUC of a host prediction at individual taxonomic levels to evaluate each feature.

Overall, the data presented are potentially promising, however in my opinion they are too limited at this point to really inform biology researchers.

i) First, the authors use AUC throughout the manuscript to evaluate the predictive power of individual features, however this metric is, in my opinion, not very informative for a researcher who would need to decide whether or not they should use these approaches in their own research. Instead, sensitivity analyses would be (I think) more useful, including some notions of how many genomes a user could expect to see a prediction for at a given accuracy, and how many hosts would remain 'unpredicted'.
ii) From a tool development perspective, one would need to understand how redundant the different features are by testing different combinations, which is not done in the current study.
iii) Hence, at this point, I feel this manuscript describes a potentially interesting set of features that may be used for host prediction, but doesn't provide enough data for a researcher to use these in their own project, or for tool developers to easily transform these into an automatic host predictor.
iv) Finally, and unfortunately, the authors also do not interpret their results from a biology standpoint beyond general descriptions such as" Some Baltimore classes are more difficult to predict than others", while more detailed interpretations would have made the paper more interesting to a broader readership.

We have addressed the four main points made above which we have numbered for ease of reference.

i) Choice of AUC as an evaluation metric.
We have included an explanation of our choice in the methods:
"Using a single metric makes it possible to compare the predictive power of the features across the large number of classifiers we tested. We have included measures of specificity and sensitivity in the supplementary tables of the results."

We have added to the discussion on the choice of which evaluation metric to optimise a classifier on may be different for different prediction tasks:
"Future development and deployment of classifiers for different virus host prediction domains would require task dependant optimisation of the models, and their operating points. Various model optimisations are possible, including combining multiple feature sets. Our results the potential of combining sets of features from different genome representations but that there is no consistent pattern as to which feature set works best for different classification tasks. Along with other model parameters, kernel weights would need to optimize the most important error metric for the task in hand(Figure 9). For example, in environmental metagenomics minimising type 1 errors -false discovery rate- is most important but when trying to identify the reservoir source of a spillover virus reducing type 2 errors is important. This optimisation should include some measure of the prevalence of the data to take account of class imbalances. For specific tasks it may also be important to test the effects of partial or incomplete genomes on the performance of the classifiers to ascertain the usefulness and robustness of these features for use in metagenomics."

On the how many virus genomes would be needed for a reliable prediction, this information can be seen in Figure 5.

ii) Feature Redundancy.
We have now included an example of how combining the kernels from different genome representations has the potential to improve prediction, and how combining kernels could be used to optimize a classifier for the evaluation metric of choice. See our methods and results sections.

iii) Usefulness to researchers developing a predictor.
The focus of this study was to compare the predictive power of a range of layered features with the potential to enhance host prediction. Our aim was not to build a tool to tackle any particular classification task. Of course, for a deployed tool, consideration would have to be given to operating points and the potential imbalance in costs for false positive and negatives. All of our features are easily generated from viral genomes and we have provided code to do so. This would enable a researcher to easily test whether or not these features would be useful in their specific domain. We have added some details in the Discussion section, on how these features could be used in the development and deployment of a predictor.

iv) Biological Interpretation.
We agree that the biological interpretation of the trends we see in our results is very interesting. The many different interpretations that are available are further confounded by issues such the number and the diversity of the viruses in the datasets. Further study to validate any conjectures is beyond the scope of this paper. Although we are wary of including unsubstantiated interpretations, we have now included in our Discussion some possible biological interpretations where we feel there is sufficient literature to substantiate our claims :

"Some Baltimore classes are easier to predict than others, for example, classifiers for both prokaryotic and eukaryotic dsDNA viruses consistently achieve higher AUC scores than RNA viruses, presumably because of the prevalence of HGT means that there are similar sequences in viruses with a shared host. Whereas all the Eukaryote RNA datasets will be affected by the fast mutation rates of leading to the loss of sequence similarity. Eukaryote classifiers for the (-)ssRNA and dsRNA viruses are significantly harder to predict than those for the (+)ssRNA viruses. This maybe due to these classes including segmented viruses. Reassortment on co-infection not only means that these viruses are highly diverse but gives them a mechanism to share genome segments across multiple hosts via subsequent co-infections."

"The lack of correlation between the size of the dataset and score is very apparent at geneus and species level, (Fig 2), with the classifiers for the datasets S.enterica(192 viruses), E.coli(465 viruses) and the genus mycobacterium(262 viruses) performing particularly badly across all feature sets. This poor performance may be more to do with the fact that these groups of viruses are highly diverses and mosaic [50] or that they contain high number of viruses that infect multiple hosts confounding any host specific signal."

I also noted some problems in the references cited, which are notably missing important tools such as PMID 27899557, 28957499, or 27153081. Given how similar they are to the approach proposed by the authors, these tools should certainly be discussed and ideally compared to the methods presented here (although the last point may be difficult since the authors do not really provide a tool per se).

The focus of this study was to compare the predictive potential of different features specifically for use in developing machine learning methods across a broad range of virus host prediction tasks.

To place this in context to other computational approaches to virus host prediction, we have added an overview of current non-machine learning methods including k-mer composition similarity comparison and have included these 3 references.

Detailed comments

l. 11-13: This seems to be inexact, VirHostMatcher recommends the use of 6-mers, and WIsH uses 8-mers. It is true though that these tools tend to use only one feature (e.g. nucleotide k-mer) instead of the multi-features approach proposed by the authors, and that's probably what should be highlighted here instead.

We have changed this to: "*focus on nucleotide features.*"

l. 24:" between infecting viruses" is a bit unclear to me, do the authors means" between viruses infecting similar hosts" ?

We have changed this to: " *between viruses infecting related hosts*"

l. 27: 'orphan' could maybe be changed to 'uncultivated' which would more specifically defined the scope of the approach proposed here ?

We have changed this to: "*metagenomically derived viruses*"

l. 52: Reference [4] seems to be incorrect, as it predates the rise of metagenomic sequence in IMG/VR. Perhaps the authors wanted to reference PMID 31120025 instead, which does reference the IMG/VR database and ~ 5% of sequences with predicted hosts ?

We have replaced this reference with *Roux (2019) for the 5% figure and for the IMG/VR databe we included Paez-Espino et al. (2018) citation.*

Figure 1: I am a bit confused by the" Feature Extraction" step in this schematic. It only mentions" K-mer composition matrix", however the authors indicate" Physio-chem properties" and" Predicted PFAM domains" in the Genome representation step before ?

To make this clearer we have changed figure 1 from "K-mer composition matrix" to "*K-mer/domain extraction.*"

The physio-chemical properties of the amino acid sequences were represented as k-mers, as described in the methods. We have re-written this section in the methods to improve clarity:

"*Physio-chemical properties* *of the amino acid sequence, k-mers (PC_k) were extracted by first binning each amino acid into one of seven groups defined by their physicochemical properties, ({AGV}, {C}, {FILP}, {MSTY}, {HNQW}, {DE}, and {KR}), [The 'PC' k-mers were then extracted using the seven bin labels as the alphabet."*

Figure 2: The authors must provide a key for the x-axis abbreviations in the legend, so that a reader can interpret the heatmap.

This has now been added to the legend.

Figure 3: This heatmap is currently missing y-axis labels (in my version), so that it is impossible to interpret in its current form.

Apologies, due to the number of rows in this heatmap and the journal restriction on the size of the figures and font size,we are unable to include row labels. We have thus included a fully annotated heatmap, including dataset sizes, in the supplementary data.

l. 159-160:" some hosts are more challenging to predict": could the authors maybe expand a little more on which host taxa are more difficult to predict, and what could be the cause of this ? I am especially puzzled by mycobacterium and synechococcus which seem to be associated with the lowest AUC, yet would also be in my opinion the only two groups for which we have enough phage genomes to robustly evaluate the authors' approach.

Looking into this we have discovered an issue in the virus-host database VHDB where some Refseqs have been reported to infect the same host at different taxonomic levels, i.e., at species rank with one taxid and at genus rank for another. We have now corrected our code to check that refseqs are only added once. Interestingly, some of the larger datasets at genus (Mycobacterium) and species level remain difficult to predict. We have now included some possible reasons for this in the Discussion:

"The lack of correlation between the size of the dataset and score is very apparent at geneus and species level, (Fig 2), with the classifiers for S.enterica (192), E.coli (465) and the genus Mycobacterium (262) performing particularly badly across all feature sets. This poor performance may be more to do with the high number of viruses that

infect multiple hosts and that these groups of viruses are both highly diverses and mosaic [45] confounding any host specific signal."

l. 184: "for, example," should be" , for example,"

This has been corrected :" *sparse, for example,"*

l. 228: Section "The predictive signal contains both phylogenetic and convergent elements." This section is interesting in its attempt at deciphering whether the classifier is trained on a phylogenetic signal (i.e. viruses of the same host look like each other) or some other / distinct host adaptation features. However, I am worried that the approach used by the authors (leaving out a family) is too coarse to be really informative, given the massive variation of phylogenetic similarity between genomes across families (as currently defined). There is also a number of misclassified genomes that could easily lead to a fake impression of a" convergent" signal while the classifier would really be based on virus-virus similarity. If the authors mean to investigate this aspect robustly, they should e.g. perform all-vs-all whole genome ANI for phages, and then hold out based on these ANI values (i.e. not including any genome pairs with ANI > XX% in the training vs test set).

We agree that using viral taxonomy as a substitute for phylogeny is incorrect but due to the lack of phylogenetic tree for all phage we hoped this would be a reasonable proxy given that in general phage genera appear to represent distinct clades of viruses. We have now included a filtering step using ANI to make sure that any viruses of greater than 75% ANI to any of the holdout viruses are removed from training.The new results have been added and a description of this is included in the Methods, Results and Discussion sections.

l. 240: "Podoviridae" and all other viral taxon names should be italicized

We have added italics.

l. 265: should" lose" be" loss" in" signal lose" ?

We have made this correction:*" The signal loss for holdout classifiers."*

l. 299: "ignoring" should be" ignore"

We have made this correction.

l. 406: "Table Supplementary X" should be replaced with one of the supplementary tables

This table has been removed as it contained redundant information.

Reviewer #3:

Thank you for your time in reviewing our manuscript and for your comments, we have addressed them all, making corrections and adding further explanations to the manuscript.

All our responses are in blue with text from the manuscript in quotes and italics, and with new or altered text in blue.

The work describes the use of SVM classifiers to compare different feature sets for their predictive power in viral host prediction. The massive increase in virome datasets makes such predictive tools an extremely important asset in the analysis of such data. The work described shows how SVM can be used to predict viral hosts. The manuscript is generally well written, providing an introduction to the research question and clear representation of the results. The methods are freely available on github

Minor comments

Line 46 – not sure reference 2 is the most appropriate. Reviews by Suttle et al (or many other reviews) better explain the importance of viruses in biogeochemical cycling

We have now included the Suttle et al (2007) reference.

P21- what was used to extract the kmers?

We used our own code to extract the k-mers from the different sequences.To improve the clarity of our methods we have:

i) added an extra sub-heading heading in methods" *K-mer extraction*"

ii) improved the description of how we generate a k-mer composition matrix:

"All sequence data was represented as vectors of k-mer composition. These were generated by counting the number of times each possible k-mer occurs within the sequence and then normalising the resulting vector to sum to 1 to account for varying lengths of the genomes."

P21 L 406 – table number missing

This table has been removed as it contained redundant information.

L410 – where any cutoffs used for domain identification ? Please state them if so

We have added the cutoff setting to the methods:

"Hmmscan settings: The --cut_tc option was used, this uses the trusted bit score thresholds from the model. The aim being to include maximum number of possible domains with the expectation that the machine learning will be able to find the true signal above the noise."

Minor issues in the references, I suspect a reference manager issue

Bacterial names to be italicised eg Synechococcus [5]

This reference about the viral tagging has been removed in response to Reviewer 1.

Inconsistent capitalisation of titles [7]

We have checked all references to remove inconsistent capitalisation.

Provision of DOIs on some references [10, 13]

We have checked all the DOIs and they all appear to be working now.

Incorrect citation of HMMER [40] cite the paper not the user guide

We have added:

Eddy SR. Accelerated Profile HMM Searches. PLOS Comput Biol. 2011;7: e1002195. doi:10.1371/journal.pcbi.1002195
