# Supplementary material for: Predicting host taxonomic information from viral genomes: A comparison of feature representations
Source: PLoS Comput Biol. 2020 May 26;16(5):e1007894. doi: 10.1371/journal.pcbi.1007894 (PMC7307784; doi:10.1371/journal.pcbi.1007894)
Supplement: Attachment — Submitted filename: Young-etal-response_to_reviewers.pdf [file pcbi.1007894.s008.pdf]

Responses to reviewers' comments.

Reviewer #2:

Thank you again for your time in reviewing our manuscript and the constructive comments. We have found them very useful and have revised the manuscript in response to your comments. In response to your more general comments we have:

- Thoroughly reviewed the manuscript removing any remaining typographic and grammar errors.
- Rewritten parts of the text to remove internal contradictions and improve clarity. The parts that have been changed substantially have been highlighted in the manuscript in blue.
- Extended all figure and table legends to allow stand alone interpretation of each and included a clearer explanation of our use of acronyms for the labels within the figures.
- Replaced Figures 10 and 11 to with new figures that more clearly reflect the new results that were added to the last revision.

Below, we have responded to all your points and have addressed the more major points individually with the new text included in this response (in blue italics) .

Point 1

p. 2: "or machine learning approaches as described below.": I disagree: machine learning are one approach used to interpret the different features mentioned by the authors, but not an alternative to e.g. "k-mer composition comparison" or "co-abundance between virus and host"

> We have added the further qualification to clarify that we are talking about the different methods as opposed to different features.
>
> *; distance based metrics of oligonucleotide or k-mer composition either with potential host genomes [7,9,10] or with a reference virus genomes [11]; or machine learning methods using a variety of sequence derived features as described below.*

Point 2

p. 2: "Machine learning approaches offers reference- and alignment-free alternative": I also disagree: machine learning approaches rely 100% on a training set, which is the equivalent of "references" in other approaches such as prophage detection

> We have re-written this to clarify that machine learning methods still rely on training examples.

Machine learning approaches offer alternatives that are not dependent on reference genomes or alignment, relying instead on a set of labeled training examples.

Point 3

p. 23: “The resulting signal will be more relevant to diverging sequences and thus this signal is more likely to have been removed in the holdout datasets”

XX → I am confused by this statement: wasn’t this the exact opposite of the holdout idea, i.e. holding out the dataset mean that most of the signal should come from diverging sequences ?

Said otherwise, a drop of AUC in holdout datasets would suggest that the signal was mostly originating from phylogenetic relationships between viruses (i.e. similar viruses), rather than host-specific features conserved across unrelated viruses infecting the same host ? I think this should be clarified in the text as this is an important point in this type of analysis.

We have re-written this paragraph to clarify our argument.

Physio-chemical features are not changed by conservative amino acid substitutions. One possible explanation for the drop in performance of PC features is that as sequences diverge, they will remain more similar at the PC level than at nucleotide and AA levels. Likewise, protein domains remain more identifiably homologous in divergent genomes, whereas convergence of domains is rare[45]. Removing the signal originating from the phylogenetic relationships between viruses in the holdout datasets may therefore lead to a larger drop in AUC for these more evolutionary-linked features. Cases where the domain signal is not lost may indicate a distant phylogenetic relationship or be due to common domains arising as a consequence of horizontal gene transfer (HGT).

Point 4

p. 27: “Reassortment on co-infection not only means that these viruses are highly diverse but gives them a mechanism to share genome segments from multiple hosts .” I am not following the logic here: co-infection means that viruses infect the same host, so how would this lead to higher rate of exchange between viruses infecting multiple hosts ? Please clarify (or remove)

We have removed this from the text.

Point 5 (Legends)

We have addressed all the following points regarding table and figure legends.

Table 1: Please specify the meaning of “PC” in the table legend.

We have added a new table legend.

Genome representation: DNA - nucleotide sequence; AA - amino acid sequence of CDS regions; PC - physio-chemical properties, each amino acid residue binned into one of seven bins based on its physio-chemical property; Domains - presence of PFAM domain in the sequence.

p. 10: “PC_5 is from the physio-chemical sequences with k-mers of length 5”. Why do the authors only mention “PC_5” instead of explaining the different acronyms (AA / PC) and then mention that the number corresponds to the k-mer size ?

same p. 16: “AA_2, is from the amino acid sequences with k-mers of length 2” ?, and for Fig. 8 legend and Fig. 9 legend

We have added a description of the feature set labels to all relevant figure legends.

The feature set labels the letters indicate the genome representation and the number the k-mer size. Genome representation:DNA - nucleotide sequence; AA - amino acid sequence of CDS regions; PC - physio-chemical properties, each amino acid residue binned into one of seven bins based on its physio-chemical property; Domains - presence of PFAM domain in the sequence.

Remaining points

For the remainder of the comments we have made all the corrections suggested and re-written our text to improve clarity.

l. 24: “Siphoviradea” should be “Siphoviridae”

Corrected

Fig. 10 needs a larger legend than “Combined kernel classifiers.”

The legend is included.

p. 28: “oligio-nucleotides” should be “oligo-nucleotides”

Corrected

p. 29: “they are wrongly labeled false” is unclear, please rephrase

Rephrased:

Secondly, the negative data are viruses that are not known to interact with the host and may include viruses for which interactions have not yet been observed, i.e., there may well be false negatives in our training/testing sets which can result in predictions incorrectly labeled as false positives.

p. 29: “may be more to do” should be “may have more to do”

Changed to : *may be due to*

p. 29: “diverses” should be “diverse”

Corrected

p. 29: “all available host labelled data available” should be rephrased to avoid the repetition

Corrected and rephrased:

While we restricted our study to using species reference sequences, a wider study using all available host labelled data from databases such as MVP database …

p. 29: “We have limited this study to using k-mer composition of the sequences” I am confused by this statement, since the authors also use PFAM domains, which seems to provide a similar (if not better) signal than k-mer in some situations ? (e.g. Fig. 2 panel A - “All”) ?

Rephrased :

In this study, we have limited the sequence composition derived features (nucleic acid, amino acid and physio-chemical properties) to fixed k-mers, not allowing mismatches.
